# Artifacts in Musculoskeletal Ultrasonography: From Physics to Clinics

**DOI:** 10.3390/diagnostics10090645

**Published:** 2020-08-27

**Authors:** Wei-Ting Wu, Ke-Vin Chang, Yu-Chun Hsu, Po-Cheng Hsu, Vincenzo Ricci, Levent Özçakar

**Affiliations:** 1 Department of Physical Medicine and Rehabilitation, National Taiwan University Hospital, Bei-Hu Branch, Taipei 10845, Taiwan; wwtaustin@yahoo.com.tw (W.-T.W.); myronrbman@gmail.com (P.-C.H.); 2 Department of Physical Medicine and Rehabilitation, National Taiwan University College of Medicine, Taipei 10048, Taiwan; 3 Department of Physical Medicine and Rehabilitation, Taipei Veterans General Hospital, Taipei 11221, Taiwan; viph062@gmail.com; 4 Department of Biomedical and Neuromotor Science, Physical and Rehabilitation Medicine Unit, Istituto di Ricovero e Cura a Carattere Scientifico Rizzoli Orthopedic Institute, 40136 Bologna, Italy; vincenzo.ricci58@gmail.com; 5 Department of Physical and Rehabilitation Medicine, Hacettepe University Medical School, Ankara 06100, Turkey; lozcakar@yahoo.com

**Keywords:** ultrasound, artifact, musculoskeletal, beam, attenuation, reflection

## Abstract

Ultrasound appears to be the most useful imaging tool in the diagnosis and guided treatment of musculoskeletal disorders. However, ultrasonography has been criticized for being user dependent. Therefore, medical professionals should be familiar with the basic principles of ultrasound imaging (e.g., physics and technical skills) to diminish artifacts and avoid misinterpretation. In this review, we focused on the physics of common artifacts, their clinical significance, and the ways to tackle them in daily practice during musculoskeletal imaging. In particular, artifacts pertaining to the focal zone, beam attenuation, path and side lobe of the beam, speed of the sound, and range ambiguity were described.

## 1. Introduction

Owing to advantages like portability, real-time assessment [1,2], and absence of radiation, high-resolution ultrasound (US) has emerged as the most useful imaging tool [3,4] in the diagnosis [5,6,7] and guided treatment [8,9] of musculoskeletal disorders. In addition to getting acquainted with scanning skills and sonoanatomy [10,11], medical professionals should also be aware of basic US physics and avoid or tackle the artifacts occurring during imaging.

**Figure 1:**
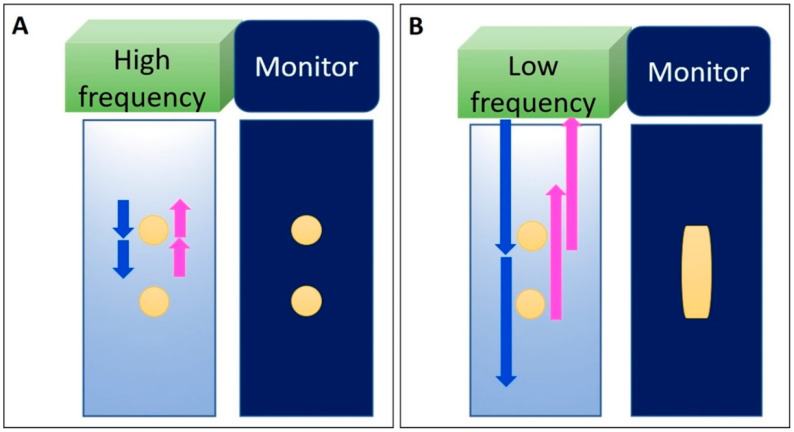
Axial resolution of the US image is related to the frequency. Two objects can be clearly differentiated when their distance is greater than half of the spatial pulse length (**A**). If the distance is smaller than half of the pulse length, the two objects cannot be differentiated during US imaging (**B**). Blue arrow: the projected US signals; pink arrow: the returned US signals. Yellow circles: the objects in the tissue or the US images of the objects insonated by high frequency US signals.Yellow column: the overlapped images derived from the two vertically aligned objects. Yellow circles: the objects in the tissue or the US images of the objects insonated by high frequency US signals. Yellow column: the overlapped images derived from the two vertically aligned objects.

In terms of basic US physics, ultrasonic waves are generated from electrical pulses through piezoelectric crystals of a transducer and then are transmitted into soft tissues through the gel [12]. US waves might be absorbed, refracted from the original trajectory, or reflected back to the transducer when reaching the interface between two different materials. The time delay between the transmitted pulse and received echo is directly related to the depth of the target shown on the monitor. The resolution of the US images is related to the transducer frequency and depth of the tissues to be visualized. Axial resolution is associated with one-half of the spatial pulse length. If the distance between the two subjects is greater than one-half of the pulse length, the two structures can be clearly differentiated (Figure 1A). On the contrary, if the distance is smaller than one-half of the pulse length, the two structures will be overlapped on US imaging (Figure 1B).

US artifacts are common while scanning for musculoskeletal pathologies and may indeed lead to incorrect diagnoses [13,14]. However, if the examiners are aware of why and when the artifacts develop, clinical misinterpretation of the US images can substantially be avoided. Furthermore, when correctly recognized, certain artifacts can even facilitate the diagnosis in clinical practice. In this review, we mainly aim to elaborate on artifacts pertaining to the focal zone, beam attenuation, path of the beam, side lobe of the beam, speed of the sound, and range ambiguity.

## 2. Improper Adjustment of the Focal Zone

### 2.1. Focal Zone Artifact

#### 2.1.1. Physics

**Figure 2:**
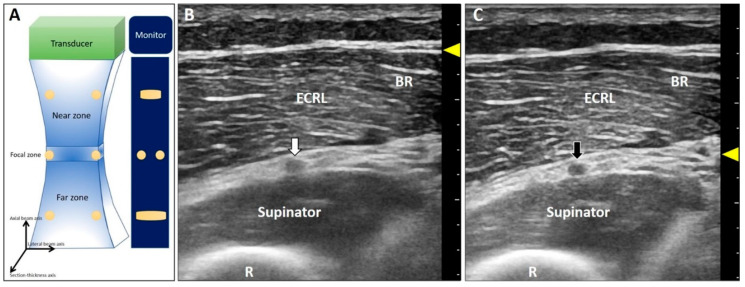
The US beam width is the narrowest at the focal zone with the best lateral resolution of two objects perpendicular to the beam (**A**). The deep radial nerve is blurred at the far zone (**B**) and becomes clearer when the focus has been set at the same depth as the nerve (**C**). White arrow: the blurred image of the deep radial nerve due to the improper location of the focal zone; black arrow: the clear image of the deep radial nerve after adjustment of the focal zone; yellow arrowheads: focal zone. ECRL: extensor carpi radialis longus muscle; BR: brachioradialis muscle; R: radius.

This artifact is also known as beam-width artifact and is associated with lateral resolution and section thickness of the US beam [14]. Beam width (Figure 2A) and section thickness are the narrowest in the focal zone. Hence, the resolution for differentiating two objects perpendicular to the propagation of US waves is the highest. If the distance between two adjacent targets is smaller than the width of the US waves, their images will be overlapped on the monitor. Accordingly, in the near or far zones, the resolution will decline because of the divergence of the US beams (Figure 2A).

#### 2.1.2. Clinical Examples

Poor resolution of a lesion makes it indistinguishable from the normal structure. For instance, concerning nerve entrapment [15], measurement of the cross-sectional area is crucial for diagnosis. If the focal zone is not placed at the right depth, the epineurium will appear blurred [16]. Another example is imprecise differentiation of the neural fascicle of the deep radial nerve from the connective tissues in the supinator tunnel when the target/focus is placed at the far field (Figure 2B). Once the focus is tuned to the suitable depth, the epineurium of the nerve is clearly presented in contrast to the adjacent tissues (Figure 2C).

## 3. Attenuation of Ultrasound Signals

### 3.1. Posterior Enhancement

#### 3.1.1. Physics

**Figure 3:**
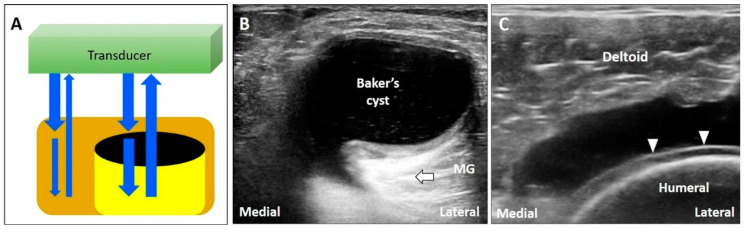
If the US beam passes through a low-attenuating structure, the signals reflected from the deep structure increase in relation to the surrounding tissues (**A**). The areas deep to a Baker’s cyst (**B**) and a complete tear of the supraspinatus tendon (**C**) become hyperechoic because of the posterior acoustic enhancement (and discrepancy of the acoustic impedance between two different tissues). White arrow: artifact due to posterior acoustic enhancement; white arrowhead: cartilage interface sign. MG: medial gastrocnemius muscle. Blue arrows: the projected and reflected US beams. Black circle: the low-attenuation structure.

The energy of US waves attenuates through absorption, reflection, or scattering. If the US beam passes through a low-attenuation structure, like effusions or cysts, the signals reflected from the deep structure increase in amplitude compared to the ones from the surrounding tissues (Figure 3A) [17]. In addition, overcompensation of tissue attenuation using the function of time-gain control on the US machine can also result in posterior acoustic enhancement.

#### 3.1.2. Clinical Examples

**Figure 4:**
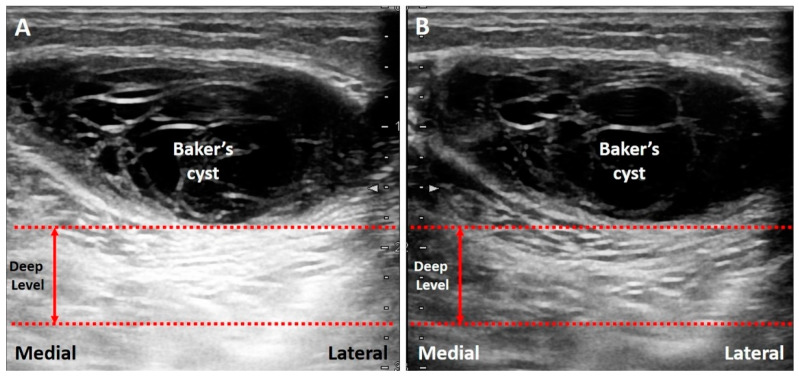
The area deep to the Baker’s cyst is hyperechoic, making it difficult to differentiate the echotexture (**A**). Decreasing the signal gain at the deep level can reduce the posterior enhancement and help in clarification of the echotexture (**B**). Red dash line: the level deep to the target. Red double arrow: the range of the depth regarding the area highly influenced by the artifact.

Common pathologies with low-attenuation features include bursitis, ganglion cysts, hematoma, abscess, and joint effusion. For instance, the Baker’s cyst (Figure 3B), communicating with the knee joint, is a common fluid-filled mass located at the popliteal fossa. It is associated with osteoarthritis, rheumatoid arthritis, and meniscus tears. Under US imaging, the anechoic mass is situated between the medial gastrocnemius muscle and the semimembranosus tendon [18]. Tissues deep in the Baker’s cyst appear hyperechoic due to posterior acoustic enhancement. Likewise, in case of a complete tear of the supraspinatus tendon [19], the torn part exhibits effusion. The surface of the humeral cartilage becomes brighter due to increased reflection of the US beam deep to the effusion and marked difference in acoustic impedance between the effusion and humeral cartilage. This presentation is also called the “cartilage interface sign” (Figure 3C) [20]. If a lesion is deep to a low-attenuation structure (Figure 4A), decreasing the gain at the deeper region can reduce posterior enhancement and improve the resolution of the target (Figure 4B).

### 3.2. Acoustic Shadowing

#### 3.2.1. Physics

**Figure 5:**
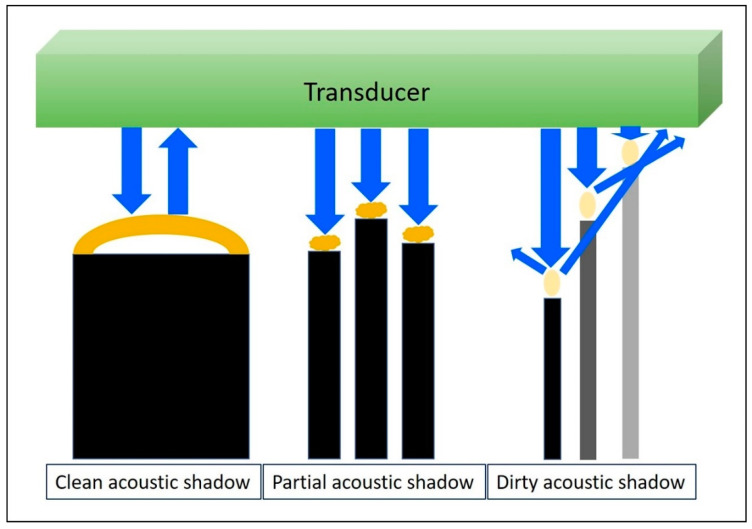
When the US wave passes through a high-attenuating structure, the echo behind the structure would be significantly reduced; forming a clean, partial, or dirty acoustic shadowing. Blue arrows: the projected and reflected US beams. Dark yellow circles: high-attenuation objects. Light yellow circles: gas bubbles.

When the US wave passes through a high-attenuation structure, like calcifications, bone [21], metal, or gas, the echo behind the structure will be significantly reduced, forming clean, partial, or dirty acoustic shadowing (Figure 5) [22]. Although US beams cannot penetrate the air, they will be reflected in multiple directions deep into the gas bubbles, resulting in a dirty acoustic shadow.

#### 3.2.2. Clinical Examples

**Figure 6:**
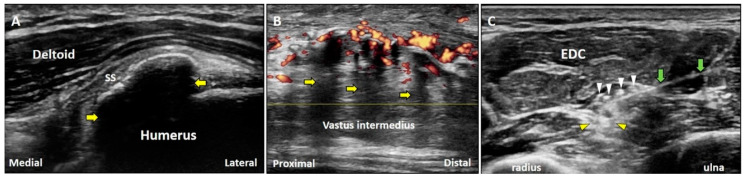
Below the large or egg-shelled calcifications, clean acoustic shadowing can be observed (**A**). Partial acoustic shadowing is observed deep to fragmented calcifications resulting from the heterotrophic ossification in rectus femoris muscle (**B**). Dirty acoustic shadowing can be observed behind the gas bubbles during an US-guided injection (**C**). Yellow arrow: acoustic shadowing artifact; white arrowhead: gas bubbles; yellow arrowhead: dirty acoustic shadowing; green arrow: needle. SS: supraspinatus tendon; EDC: extensor digitorum communis muscle.

Clean acoustic shadowing appears as an anechoic band deep in an egg-shelled calcification inside the tendon (Figure 6A). Partial acoustic shadowing can be seen as a hypoechoic region deep inside fragmented calcifications (e.g., heterotrophic ossification) (Figure 6B). Dirty acoustic shadowing, presented with heterogeneous echoes within a region of low signal intensity, can be observed behind gas bubbles introduced by injections (Figure 6C). If the target is deep into a high-attenuation structure, scanning through the lateral aspect of the structure can circumvent acoustic shadowing to improve the visibility of the target.

### 3.3. Reverberation

#### 3.3.1. Physics

**Figure 7:**
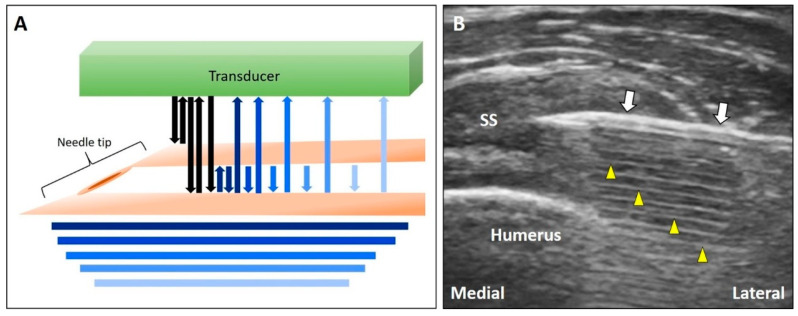
Schematic drawing illustrates multiple reverberations occurring between the two closely reflective interfaces (**A**). The comet tail appearance can be observed deep to the needle (**B**). White arrow: needle; yellow arrowhead: reverberation artifact. SS: supraspinatus tendon. Blue and black Arrows: the projected and reflected US beams.

When US waves pass through two approximated reflective planes, they will be reflected repeatedly between the two interfaces. Part of the US signals return to the transducer, and part of them bounces back and forth between the two surfaces, forming multiple parallel lines deep into the target. These lines become shorter and less echogenic (resembling a comet’s tail) at the deeper region because of energy attenuation as they move further from the two reflective surfaces (Figure 7A) [23].

#### 3.3.2. Clinical Examples

Needle shafts (Figure 7B) or metal implants can cause the reverberation artifact. This finding is useful for the identification of the needle tip during guided injections [24]. If the target is deep into the reverberation-affected area, adjusting the angle of insonation to be more oblique to the reflective plane can circumvent this artifact.

### 3.4. Ring Down Artifact

#### 3.4.1. Physics

**Figure 8:**
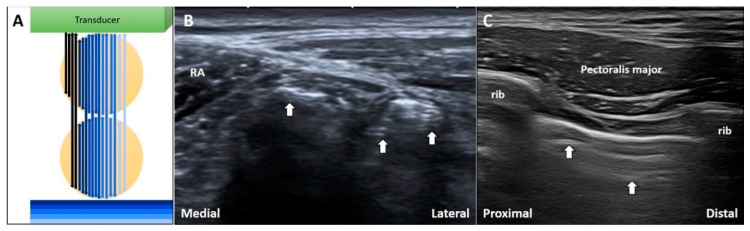
The schematic drawing illustrates the ring-down artifacts when the US waves pass through the air bubbles (**A**). During imaging for inguinal hernia, the ring-down artifacts can be seen in the bowels (**B**). During intercostal block, the ring-down artifacts can be visualized deep to the pleura (**C**). White arrow: ring-down artifacts. RA: *rectus abdominis.* Black and blue arrow: the projected and reflected US beams.

When the US beam passes through air bubbles, sound waves resonate within the bubbles, emitting continuous waves back to the transducer. This is known as the ring down artifact. The US streaks decay as they move away from the target (Figure 8A) [25].

#### 3.4.2. Clinical Examples

During investigation of an inguinal hernia, ring down artifacts are commonly observed in the bowel due to intra-lumen gas (Figure 8B). In cases of subcutaneous infections, the ring down artifact can develop through accumulation of gas formed by anaerobic bacteria. Further, during the performance of intercostal nerve block [26] or injections in trigger points of the rhomboid muscle, medical professionals should be cautious so as not to puncture the pleura that also present ring down artifacts (Figure 8C).

### 3.5. Mirror Image Artifact

#### 3.5.1. Physics

**Figure 9:**
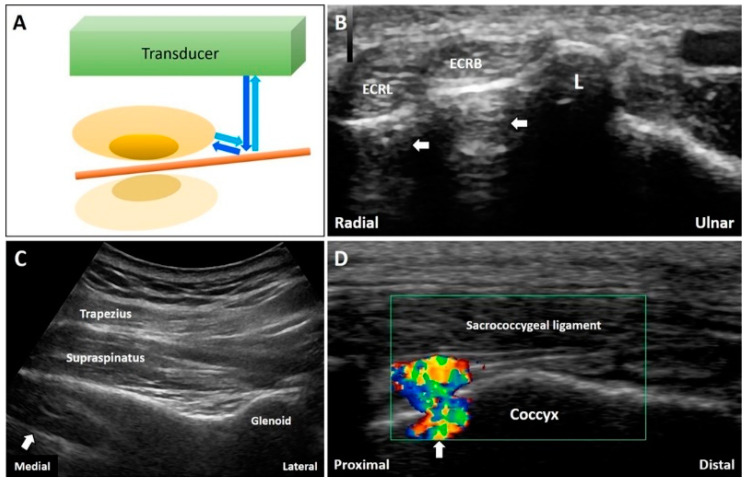
When the sound waves encounter a reflective interface, the reflected beam would cast a mirror image at the opposite side of the interface (**A**). The mirror artifacts can be seen at many body regions like the forearm (**B**), supraspinatus fossa (**C**)**,** and coccyx (**D**). White arrow: mirror artifact. ECRL: extensor carpi radialis longus tendon; ECRB: extensor carpi radialis brevis tendon; L: Lister’s tubercle Blue arrows: the projected and reflected US beams. Brown pillar: the reflective interface. Green box: the color box for detection of Doppler signals.

When sound waves pass through a target, and some are reflected to form the primary image. If the residual waves encounter an interface between two materials with distinct acoustic impedance, part of the waves are further reflected backwards. The reflected US beam hits the target and then returns to the transducer, forming a secondary image at the opposite side of the reflective interface [27]. The phenomenon is known as the mirror image artifact (Figure 9A) [28]. The target must be close to the reflective interface; otherwise, it is difficult to recognize the secondary image due to attenuation.

#### 3.5.2. Clinical Examples

The mirror artifacts are commonly seen when the target is beside a smooth reflective surface, such as a long bone. For example, during the visualization of the extensor carpi radialis longus and brevis tendons in the short axis at the distal forearm, two echogenic reflections can be seen deep in the underlying cortical bone (Figure 9B). When a suprascapular nerve block is performed [29], the mirror artifacts of the supraspinatus muscle can be observed symmetrically on the other side of the scapular spine (Figure 9C). Artifacts can also be visualized when the Doppler mode is turned on to track a vessel overlying a bony cortex (Figure 9D). Of note, decreasing the gain deep to the reflective surface can reduce the artifact.

### 3.6. Anisotropy

#### 3.6.1. Physics

**Figure 10:**
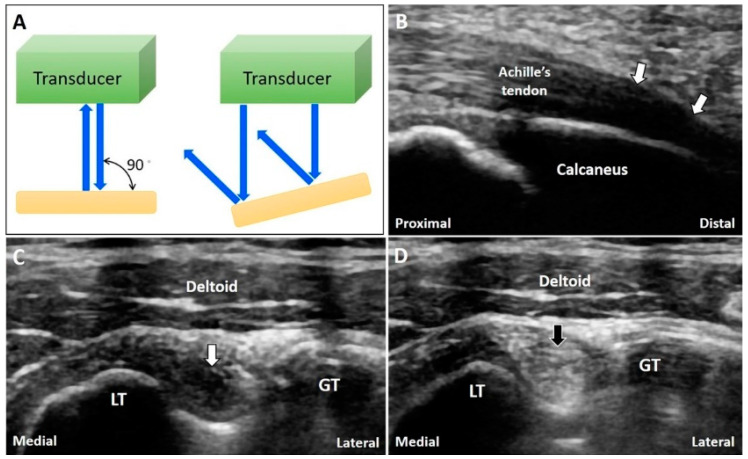
The US beam is totally reflected back when the transducer is perpendicular to the target while they are reflected away from the transducer if the object is not perpendicular to the US beam (**A**). The anisotropy is seen at the insertion of the Achilles tendon (**B**). A normal biceps long head tendon can be misinterpreted as tendinopathy due to anisotrophy (**C**) and tilting the transducer to make it perpendicular to the bicipital groove can eliminate the artifact (**D**). White arrow: anisotropy artifact; black arrow: image after compensation. GT: greater tubercle; LT: lesser tubercle. Blue arrows: the projected and reflected US beams.

The artifact of anisotropy results from the angle of insonation and can be observed when fibrillary linear structures, such as tendons, ligaments, and nerves, are scanned. If the US beam is not perpendicular to the target, the signals will be reflected away from the transducer, making the target look hypoechoic (Figure 10A) [30].

#### 3.6.2. Clinical Examples

The tendons or ligaments are not always parallel to the skin. Due to anisotropy, they may appear hypoechoic and thus resemble tendinosis or tears. For instance, the enthesis of the Achilles tendon in the long-axis view usually appears hypoechoic [31] due to the angle of insonation (Figure 10B); hence, the artifact can be reduced by light compression at the distal end of the transducer. Moreover, a normal long head of the biceps tendon can be misinterpreted as tendinopathy due to anisotropy (Figure 10C). Tilting the transducer to make it perpendicular to the bicipital groove can eliminate this artifact (Figure 10D).

### 3.7. Side Lobe Artifact

#### 3.7.1. Physics

**Figure 11:**
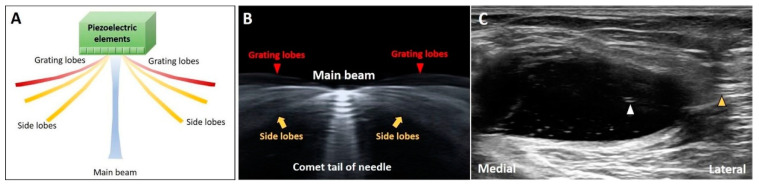
Schematic drawing illustrates the directions of the central beam and lateral dispersion of residual off-axis beams (side and grating lobes) (**A**). The lateral dispersion of US beams can be clearly observed with a lot of coupling agents in between the needle and transducer (**B**). The side lobe artifact can be observed inside the cystic lesion (**C**). Yellow arrow: side lobe; red arrowheads: grating lobes; white arrowhead: nail plate. White arrowhead: the ghost image on the main axis of the US beam. Yellow arrowhead: the object on the path of the side lobe that causes the side lobe artifact.

Most of the US energy is transmitted through the central beam. Because of the radial expansion and compression of the transducer’s crystal elements, lateral dispersion of residual off-axis beams (side and grating lobes) can be observed (Figure 11A). The artifacts can be easily seen when numerous coupling agents (gel or water) accumulate between the target and transducer (Figure 11B). When the US beam encounters an object and then returns to the transducer, the US machine misinterprets the signals from the side lobes as waves originating from the main beam, projecting a “ghost image” on the main axis [13,32].

#### 3.7.2. Clinical Examples

While investigating a cystic lesion, we can occasionally visualize certain hyper-reflective objects inside the cyst. The hyper-reflective images might be derived from the structures/tissues located on the path of the side lobes, which would be mistaken as the targets on the main axis of the US beams. (Figure 11C). The investigators are suggested to slightly adjust the position of the transducer to examine whether the hyper-reflective images consistently exist.

## 4. Speed of the Sound

### 4.1. Refraction Artifact

#### 4.1.1. Physics

**Figure 12:**
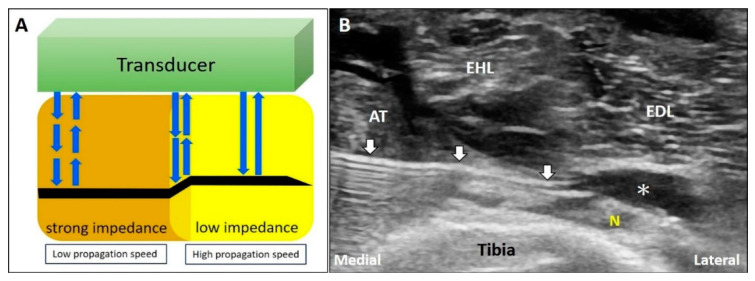
When the US beam travels through an area with strong impedance, the delayed return of the US signals to the transducer would lead to overestimation of the depth of the object. In contrast, if the target is located in the area of low acoustic impedance, the object shown on the monitor would appear shallower that its actual depth (**A**). During deep peroneal (fibular) nerve block; as the propagation speed of sound waves in the muscle is faster than that in the fat, the needle shaft in the fat pad will be seen bended toward the tibia bone (**B**). White arrow: refraction artifact; asterisks: injectate. AT: tibialis anterior muscle; EHL: extensor hallucis longus muscle; EDL: extensor digitorum longus muscle; N: deep peroneal (fibular) nerve. Blue arrows: the projected and reflected US beams.

The speed of sound varies across tissues with different acoustic impedance. When the US beam travels through an area with strong impedance, the delayed return of the US signals to the transducer leads to overestimation of the depth of the object. In contrast, if the target is located in an area of low acoustic impedance, the object shown on the monitor appears shallower than it actually is (Figure 12A) [33].

#### 4.1.2. Clinical Examples

During US-guided injection, if the needle pierces two areas with different acoustic impedance, it will look bended due to refraction. During deep peroneal (fibular) nerve block, the needle pierces the interface between the tibialis anterior muscle [34] and the fat pad surrounding the nerve (Figure 12B). As the propagation speed of sound in the muscle (1580 m/s) is faster than that in the fat tissue (1450 m/s), the needle shaft in the fat pad will appear to be bended towards the tibia.

### 4.2. Edge Artifact

#### 4.2.1. Physics

**Figure 13:**
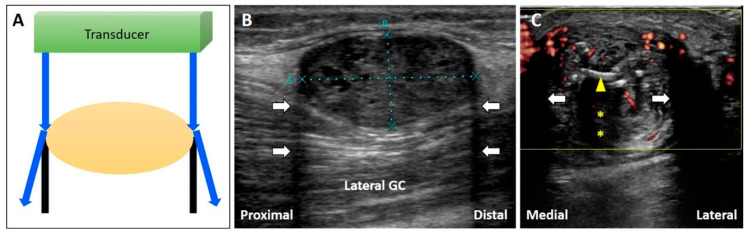
Schematic drawing illustrates the hypoechoic parallel lines projecting along the edges of the curved structure because most of the US beam is reflected away from the transducer (**A**). The edge artifact can be observed at the edges of a circular structure, such as a schwannoma of the lateral sural cutaneous nerve (**B**). This artifact may be misinterpreted as thickened Achilles paratenon (**C**). White arrow: edge artifact; yellow arrowhead: sutures; yellow asterisk: acoustic shadowing due to sutures. GC: gastrocnemius muscle.

When the US beam hits the edge of a curved surface, most of the US beam is reflected away from the transducer, appearing as hypoechoic parallel lines projecting along the edges of the target (Figure 13A).

#### 4.2.2. Clinical Examples

This artifact can be observed at the edge of a circular structure, such as a schwannoma of the lateral sural cutaneous nerve (Figure 13B). During scanning of a patient with Achilles tendinopathy (Figure 13C), the artifact may be misinterpreted as a thickened paratenon. Adjusting the angle of insonation (e.g., heel-toe maneuver) can circumvent the artifact.

### 4.3. Range Ambiguity Artifact

#### 4.3.1. Physics

**Figure 14:**
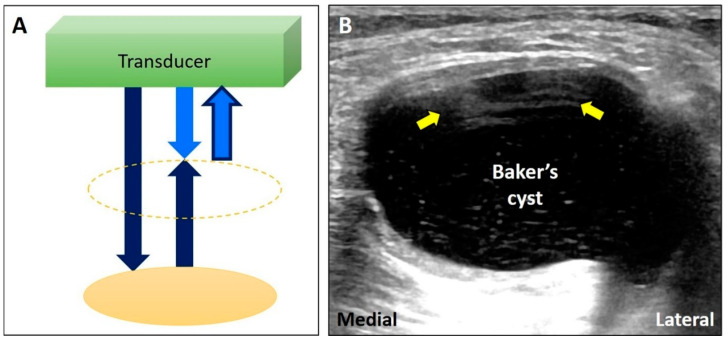
Schematic drawing illustrates that the first US pulse (dark blue arrow) hits the object and returns to the transducer after the second pulse (bright blue arrow) is projected. The US machine misrecognizes the first returning pulse as the second returning pulse (bright blue arrow with dark blue frame). A ‘ghost’ object will appear at a more superficial level because its depth is underestimated (**A**). US imaging of a huge Baker’s cyst is accompanied by the artifact as intracystic septae because of the echoes from the deep wall of the cyst (**B**). Yellow arrow: range ambiguity artifact.

The first US pulse hits an object and returns to the transducer after the second pulse is projected. The US machine misrecognizes the first returning pulse as the second returning pulse. Therefore, the time interval between emitting and receiving a pulse is erroneously shortened. Consequently, a “ghost object” will appear at a more superficial level than where the object is truly located (Figure 14A) [14].

#### 4.3.2. Clinical Examples

If the target is a big cyst, it takes a longer time for the transducer to receive the reflected echo from the deep wall of the cyst. When the first pulse from the posterior wall returns to the transducer, the second pulse has already been projected, forming several parallel lines inside the cyst (especially superficially) resembling intra-cystic septa (Figure 14B). Decreasing the pulse repetition frequency can reduce the range ambiguity artifact.

## 5. Conclusions

Artifacts are commonplace in musculoskeletal US imaging. Some of them can be reduced by adjusting the transducer position and/or machine settings. As far as the rest are concerned, medical professionals must be aware of their mechanisms and the possibility of relevant fallacious diagnoses. Importantly, it is noteworthy that prompt recognition of these artifacts will contribute to more accurate and easier diagnoses and interventions alike.
